# Classification of Alzheimer’s Patients through Ubiquitous Computing †

**DOI:** 10.3390/s17071679

**Published:** 2017-07-21

**Authors:** Alicia Nieto-Reyes, Rafael Duque, José Luis Montaña, Carmen Lage

**Affiliations:** 1 Department of Mathematics, Statistics and Computer Science, Universidad de Cantabria, 39005 Santander, Spain; rafael.duque@unican.es (R.D.); joseluis.montana@unican.es (J.L.M.); 2 Cognitive Disorders Unit, Department of Neurology, Marqués de Valdecilla University Hospital (HUMV) Valdecilla Biomedical Research Institute (IDIVAL), 39008 Santander, Spain; clage@idival.org

**Keywords:** Alzheimer, functional data analysis, healthcare, hypothesis testing, pattern recognition, supervised classification, ubiquitous computing

## Abstract

Functional data analysis and artificial neural networks are the building blocks of the proposed methodology that distinguishes the movement patterns among c’s patients on different stages of the disease and classifies new patients to their appropriate stage of the disease. The movement patterns are obtained by the accelerometer device of android smartphones that the patients carry while moving freely. The proposed methodology is relevant in that it is flexible on the type of data to which it is applied. To exemplify that, it is analyzed a novel real three-dimensional functional dataset where each datum is observed in a different time domain. Not only is it observed on a difference frequency but also the domain of each datum has different length. The obtained classification success rate of 83% indicates the potential of the proposed methodology.

## 1. Motivation and Research Context

Due to the impact of the ubiquitous computing paradigm, the desktop computer has given way to smaller devices (smartphones, tablets, etc.), body computers (bracelets, spectacles for augmented reality, etc.) and near field communication/radio frequency identification (NFC/RFID) cards embedded in objects of the user’s environment [1]. Many of these new computers can collect information about the user activity without having to make any interaction efforts (for example, smartphones collect GPS information of the users, NFC/RFID technology identifies people who access to restricted areas, etc.). This new paradigm has a great impact in the field of healthcare due to these small computers can easily monitor the patient’s activity to know more in depth the effects of the disease [2].

Several neurological disorders are accompanied by difficulties in performing movements. In particular, dementia constitutes a progressive condition characterized by cognitive impairment and frequently associated with movement disorders. Alzheimer’s disease, as the leading cause of dementia, is a neurodegenerative disease that has become a major concern to public health due to the economic and social burden that it means. The severity of the disease is usually established by clinical assessment and based on the patient’s ability to perform activities of daily living (ADLs). In clinical practice, the most used assessment tool is the Global Deterioration Scale [3], which includes different stages: no objective cognitive impairment (GDS 1-2); mild cognitive impairment when there is no significant functional impact (GDS 3); and dementia when the cognitive impairment leads to some functional impairment (mild dementia or GDS 4, moderate dementia or GDS 5, moderately severe dementia or GDS 6 and severe dementia or GDS 7).

According to the current literature, Alzheimer’s disease is linked to specific walking patterns which make possible to distinguish between these patients and healthy subjects. Additionally, some of these motion abnormalities are present since very early stages of the disease, which means that their analysis may have an important diagnostic value in prodromal stages, when the evolution of a patient suffering from memory problems is still unknown. However, these abnormalities are not appreciable by the human eye, so it can be necessary to use an instrumentalized assessment such as accelerometry to provide detailed data of the movement features of the patients.

Moreover, patients who have already been diagnosed can be favored by devices that analyze their movements (gyroscopes, accelerometers, GPS, etc.) to assess how the evolution of the pathology affects their mobility. Several studies have analyzed the relationship between Alzheimer’s disease and mobility problems by means of devices that automatically extract data on the user’s movements. These studies have focused on very specific tasks such as walking a certain distance by subjects that wear body computers. However, few works have researched whether an analysis of daily activities using personal smartphones may have value for diagnosing and monitoring the pathology. This type of analysis would involve processing a set of important data since the subject would be constantly monitored performing tasks of different nature.

We propose and experiment with a methodology that studies the relationship between data recorded by smartphone accelerometers in patients with Alzheimer’s disease and their daily mobility patterns. Previously, several hypothesis tests from the functional data analysis determined if there is a relationship between the stage of the disease and the patient’s mobility patterns. Then, this methodology addresses the extraction of features that enable us to reduce the size of the sample due to the smartphone records several data on the mobility of the subject by second (s). A neuronal network is automatically learned from these significant features. The main purpose of this neural network is classifying the stage of the patients’ pathology from their mobility data. Finally, 35 patients participate in a case study that put the methodology into action. The results of applying methodology in this case study are compared with other machine learning classifiers. The methodology proposed in this paper is novel in that it involves two agents:

- The patient and/or carer that turns on and off the accelerometer of the smartphone in order for it to save and analyze the data.
- The doctor that receives the analysis of the data and that has to take the decision to whether make a more thorough examination in a control ambient with other techniques.

Thus, what we propose here is a different type of analysis from what it is used at the present time with Alzheimer’s patients, and that does not substitute it but amplifies it. Here, veridical real data are analyzed. On the one hand, this implies that the data are recorded in an uncontrolled ambient and, on the other hand, that the data are collected cheaply and therefore as frequently as desired. A further implication is that the length of time during which each recording is made varies from one another.

The remainder of this article is organized as follows. Section 2 describes a case study in which we record data of the mobility of 35 patients with Alzheimer’s disease. Section 3 presents a study that uses functional data analysis to investigate the relationship between the mobility of patients with Alzheimer’s disease and the stage of this disorder. Section 4 describes a methodology to analyze the mobility patterns in patients with Alzheimer’s from data recorded by accelerometers of smartphones and the diagnosis and evolution of this disorder. Section 5 reviews the main research contributions related to our work. Section 6 analyzes the conclusions of this research work.

## 2. Real Data

The real data studied in this paper consist of the recordings of the accelerations of a sample of 35 patients with Alzheimer’s disease while they move in a daycare center. The main goal of this work is to apply a supervised learning process to determinate the stage of the disease from accelerometer data. Supervised learning is the machine learning task that infers a function from labelled training data. Therefore, the first step of this supervised learning process is to generate labelled data. The Global Deterioration Scale is the tool used to establish each label as the the stage of dementia in each patient. The assessment was conducted by the neuropsychologist of the daycare facility and it was based on the medical reports provided, the patient’s clinical examination and a clinical interview with the main carer to investigate the patient’s functional performance in his/her daily life, which altogether allowed to establish the disease stage. The Global Deterioration Scale comprises seven stages (from 1—No cognitive impairment to 7—Severe dementia). For methodological purposes, these levels were further grouped into three categories: early stage (GDS 2 and 3), middle stage (GDS 4 and 5) and late stage (GDS 6 and 7). The 35 patients were thereby categorized into three groups: early stage when they were in GDS 2 and 3 (7 patients), middle stage when GDS 4 and 5 (18 patients) and late stage when GDS 6 and 7 (10 patients). No patient in GDS 1 was included as this stage corresponds to healthy subjects.

The recordings of accelerations were obtained by the use of the accelerometer device of an Android smartphone that the patient carried in one of their pockets. The patients moved in a room of the daycare center under the supervision of the neuropsychologist. Therefore, we introduce a more flexible environment than previous works [4]. We have, however, a moderate degree of control and supervision of the patients’ activities, due to the neuropsychologist was present during the activities and all the patients were in the same room of the day-center facility, which is a context that reduces the possibilities of performing a high variety of activities. The neuropsychologist was the person responsible to place and orient the smartphone inside a small pocket of each patient. The variety of the patients’ activities in addition to any mistake performed by the neuropsychologist, to orient and place the smartphone in the pocket in the same way for all the patients, and an accidentally change of the orientation of the accelerometer are part of the study and are acceptable in samples having a meaningful size; so, we do not take any additional methodological indication for this purpose.

**Figure 1:**
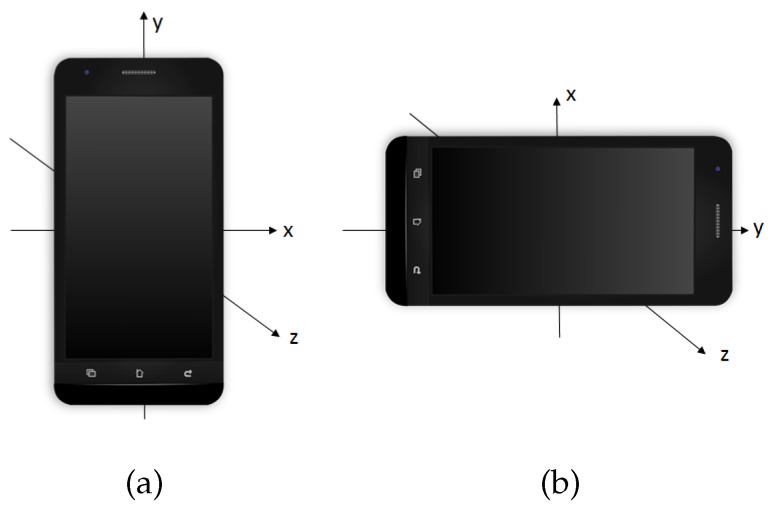
Representation of the three axes into which the accelerations are measured; and shown in two different positions, panels (**a**) and (**b**).

This type of accelerometer records the acceleration forces, of the patient carrying the smartphone, on the three spatial axes (see Figure 1) in meter/second${}^{2}$ (m/s${}^{2}$, the International System of Units). Note that these acceleration forces are the result of the gravity force together with the accelerations forces applied by the patient to generate movements such as walking. The data are easily accessible because the accelerometer device of an Android smartphone allows saving the measurements in a text file. The accelerometer takes measures of the acceleration in a range of ±3.27 g and uses a sampling rate of 8 Hz. All participants of this case study used the same model of smartphone. Most current smartphones with accelerometer enable us to use the same range of G and interpolate to a common sampling rate. Therefore, the results of this experiment are not completely dependent on the model of smartphone used by the participants.

The accelerations of the movements of each studied patient were recorded during separate periods of time for approximately a week, with no more than a recording per day. Thus, number of recordings per patient varies in the dataset from two to eight, with a total of 187 recordings among the 35 patients. In this paper, we denote by $\left\{o_{1},\ldots ,o_{n}\right\}$ with n=187 the set of measurements provided by the accelerometer carried by the Alzheimer’s patients. There, $o_{i}:=o_{i}\left(t\right)$ for i=1,…,n whith *t* denoting the time. Accelerometers does not provide the measurements for each time t, not providing the acceleration in a continuous manner. Thus, for each i=1,…,n, $o_{i}\left(t\right)$ is recorded on a grid of time-points $\{i_{1},\ldots ,i_{t_{i}}\},$ which is natural when recording real data through time.

This kind of data is known as functional data. However, the data studied in this paper have a richer topology than the usual data studied in functional data analysis. One of the reasons is that the length of the time each separate period was recorded, $|i_{t_{i}}-i_{1}|,$ varies for each of the 187 recordings.

The reason for each of the time lengths to differ is that they solely depends on when the patient and/or carer decide to turn the device on and off. Thus, the times to collect the data are not previously selected; they are just set on the spur of the moment. Of course, there are two limits for the time length: the limit of the battery of the used smartphone and the memory capacity of it, as the data are saved in it as text file.

**Figure 2:**
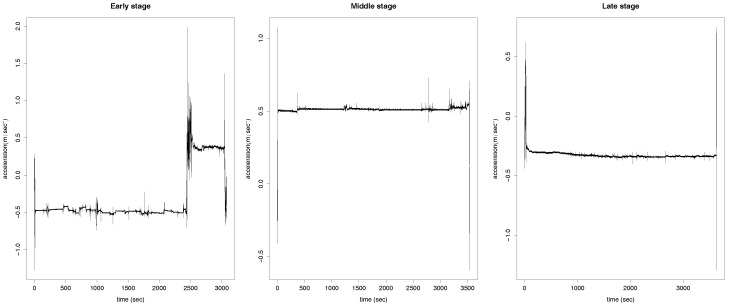
Measured acceleration on the *x*-axis for the accelerations of the patients in m/s${}^{2}$ versus the time in s, at different stages of the disease: early (**left**), middle (**central**) and late (**right**).

The difference of the time length can be observed from Figure 2, where the accelerations, on the *x*-axis, measured on their respective recorded times are displayed for three of the 187 cases. The time length on the left panel of the plot is clearly shorter than the other two. Additionally, the grid in which the data were recorded varies for each of the 187 recordings and it also varies the frequency of the measurements stored. However, each recording always contains several measurements per s. In order for the 187 recordings to be comparable, a preprocess of the data is required. We do this preprocess and the consecutive analysis of the data under two different frameworks: Functional Data Analysis (Section 3) and Machine Learning (Section 4).

Furthermore, in this paper, we additionally use the notation $o_{i}\left(t\right):=\left(x_{i}\left(t\right),y_{i}\left(t\right),z_{i}\left(t\right)\right),$ where $x_{i}\left(t\right),$ $y_{i}\left(t\right)$ and $z_{i}\left(t\right)$, respectively, are the accelerations at time *t* in each of the three spatial coordinates axis. In particular, as mentioned above, in Figure 2 it is plotted the *x* coordinate for three different patients during one of the period of times in which their accelerations are measured. The accelerometer device of the Android smartphone also computes the euclidean norm of $o_{i}\left(t\right),$ for $t\in \{i_{1},\ldots ,i_{t_{i}}\}.$ This is a redundant measure and, therefore, we do not include it in the analysis explicitly. It is worth saying that the three patients corresponding to the accelerations shown in the figure are at different stages of the disease, which is not not possible to depict by the eye. To categorize the stage of the disease of the 35 patients, we divide them into those who are in the early stage of the disease (seven patients), in the middle stage (18 patients) and in the late stage (10 patients).

Finally, we would like to focus on the fact that these real data are veridical real data recorded by laypeople. As commented in Section 1, this implies that a further analysis has to be performed by the doctor to provide an accurate diagnosis as, among other facts, the accelerometer in the smartphone is not calibrated, different smartphones are used by different patients and, sometimes, even by the same patient, and the coordinate axis of the accelerometer in the smartphone are not absolute. More important, however, is that it has the great advantage that it can be cheaply recorded as much data as desired providing high accuracy of the classification results.

Given a fixed external system of coordinates, a characteristic of the accelerometer of the smartphone is that, when it is moved, the three spatial axis change with respect to the fixed external system. Moreover, as each patient carries the smartphone in his/her pocket in a different manner, the position of the axis of the smartphone also differs from the above mentioned external axis in a different manner. In fact, not only do these differences exist among patients but also among different recordings of the same patient. This fact enriches the study that is here performed from the data analysis point of view in the sense that the three-coordinate axis are far from being three independent variables.

## 3. Functional Data Analysis

In this section, we study the data under the framework of functional data analysis. As commented in Section 2 these data need preprocessing, as each datum is measured in a different grid of time-points and for a different time length. The first is an ordinary preprocess in functional data analysis but not the second. For the first, we interpolate the data and record the resulting values at each s. That is, for each i=1,…,n, we record $a_{i}\left(t\right):=\left(x_{i}\left(t\right),y_{i}\left(t\right),z_{i}\left(t\right)\right)$ for *t* the natural numbers until the round of $i_{t_{i}},$ which we denote by $T_{i}.$

The second preprocess step regards the issue that $i_{t_{i}}$ is different for each i=1,…,n. This is not a major issue here, however, due to this data fall into the framework of stationary processes. That is, it is equivalent to start recording the data at any time *t* and therefore to obtain a set of curves measured for the same time length it suffices to do the following. Setting *l* to be the length of the curve with smallest time length amongst the n, we draw at random $l_{i}$ with the discrete uniform distribution in the set of integer numbers $\left\{0,\ldots ,T_{i}-l\right\}$ for i=1,…,n and apply our functional data analysis to $a_{i}\left(t\right):=\left(x_{i}\left(t\right),y_{i}\left(t\right),z_{i}\left(t\right)\right)$ for $t=1+{ł}_{i},\ldots ,l+l_{i}$ for i=1,…,n. To obtain results that are not influence by the random draw of the $l_{i}$’s, we repeat the analysis for 1000 times in Section 3.1 and Section 3.2 and 10 time sin Section 3.3; denoting the random draws by $l_{i}\left(j\right),$ i=1,…,n and j=1,…,1000 (Section 3.1 and Section 3.2) or j=1,…,10 (Section 3.3).

### 3.1. Functional Analysis of Variance

In this section, we propose to test the null hypothesis that the mean of the accelerations of the early-stage patients is equal to the mean of the middle-stage patients and equal to the late-stage patients against the alternative that at least one of these means is different. Thus, given three populations, $\Pi_{1}$ (early-stage), $\Pi_{2}$ (middle-stage) and $\Pi_{3}$ (late-stage), this is a test of

$$H_{0}:\mu_{1}=\mu_{2}=\mu_{3}$$

versus $H_{a}:$ there exist *i* and *j* such that $\mu_{i}\neq \mu_{j}$ with i,j∈{1,2,3}. $\mu_{1},$ $\mu_{2}$ and $\mu_{3}$ denote the respective means of the populations, i.e., $\mu_{1}$ denotes the mean acceleration of the early-stage patients, $\mu_{2}$ the mean acceleration of the middle-stage patients and $\mu_{3}$ the mean acceleration of the late-stage patients. The aim in performing this hypothesis test is to check whether the difference in means among the three sets are significant. Having differences in mean gives grounds for the possibility of developing a supervised classification procedure to assign patients to one of the three groups.

There are two main common statistics for testing one-dimensional ANOVA, one based on the $L_{2}$ norm and another in what is known as F-statistic. First, we use here three different functional analysis of variance tests, functional ANOVA, that are based on the one-dimensional F-test. Additionally, two of these tests use random projections [5] and the other the maximum of the F-statistics [6]. We summarize these tests in what follows. For that, let us assume we have three functional datasets, $r:=\{r_{1},\ldots ,r_{n}\},$ $s:=\{s_{1},\ldots ,s_{n}\}$ and $u:=\{u_{1},\ldots ,u_{m}\}$, respectively, from populations $\Pi_{1},$ $\Pi_{2}$ and $\Pi_{3};$, all of functions supported on a compact interval I.

**Functional ANOVA based on random projections.** The idea here is to do k≥1 random projections of the functional data into one-dimensional data and apply the one-dimensional F-test to each of those one-dimensional projections, obtaining *k* *p*-values. Those *k* *p*-values are combined either using the Bonferroni correction or the false discovery rate (FDR). The F-test used in [5] is parametric and assumes the Gaussianity and homoscedasticity of the random projections. Thus, we do so here, although a non-parametric version of it can be obtained by doing bootstrap, as we do in the other tests proposed in this section. Furthermore, we take k=30 and the random functions in which to project the functional data is generated using a uniform distribution in the sphere corresponding to a space with dimension the number of elements in the grid in which the functional data are observed.

**Functional ANOVA based on the F-statistics.** The idea here is to apply the one-dimensional F-test at the time coordinate in which there exists the largest difference in mean among the three groups of patients. Denoting by F(r(t),s(t),u(t)) the one-dimensional statistic at time t, the test statistic is $T\left(r,s,u\right):=\sup_{t\in I}F\left(r\left(t\right),s\left(t\right),u\left(t\right)\right).$ Here, we use a non-parametric test, using bootstrap to emulate the distribution of the data. Thus, it is computed *B* bootstrap samples from r, *s* and *u* independently. For b=1,…,B, we denote these bootstrap samples by $r_{b}^{*}:=\left\{r_{b,1}^{*},\ldots ,r_{b,n}^{*}\right\},$ $s_{b}^{*}:=\left\{s_{b,1}^{*},\ldots ,s_{b,n}^{*}\right\}$ and $u_{b}^{*}:=\left\{u_{b,1}^{*},\ldots ,u_{b,m}^{*}\right\}$ and the respective bootstrap statistic under the null hypothesis is $T(r_{b}^{*}-\bar{r},s_{b}^{*}-\bar{s},u_{b}^{*}-\bar{u}),$ where $\bar{r}$ denotes the sample mean of $\left\{r_{1},\ldots ,r_{n}\right\}$ and equivalently for $\bar{s}$ and $\bar{u}.$ Then, the *p*-value of the test is computed as $\left(1+\sum_{b=1}^{B}I\left(T\left(r_{b}^{*}-\bar{r},s_{b}^{*}-\bar{s},u_{b}^{*}-\bar{u}\right)\geq T\left(r,s,u\right)\right)\right)/\left(B+1\right)$ where I(·) is the indicator function that takes value one if $T\left(r_{b}^{*}-\bar{r},s_{b}^{*}-\bar{s},u_{b}^{*}-\bar{u}\right)\geq T\left(r,s,u\right)$ and zero otherwise. Here, we use B=999.

In the literature, there exist three major ways to construct a functional test of means as there are three main manners of applying a one-dimensional statistic to a functional data: (i) using random projections we reduce each functional datum to a one-dimensional datum by projecting it into a random function; (ii) looking for the coordinate that provides more difference amongst the means implies that we end up applying the one-dimensional statistic only on that coordinate, which leaves in a one-dimensional space; and (iii) applying the one-dimensional statistic at each time point and integrate those values over the time domain. We exemplify this third setting, using the hypothesis test whose statistic is based on the difference on $L_{2}$ norm of the means [7]. This test, however, only applies when we have two groups. Thus, we apply it separately for testing $H_{0}:\mu_{1}=\mu_{2}$ against $H_{a}:\mu_{1}\neq \mu_{2},$ $H_{0}:\mu_{1}=\mu_{3}$ against $H_{a}:\mu_{1}\neq \mu_{3}$ and $H_{0}:\mu_{2}=\mu_{3}$ against $H_{a}:\mu_{2}\neq \mu_{3}.$

**Test of equality of means based on the $L_{2}$ norm.** Given two populations $\Pi_{1}$ and $\Pi_{2},$ this is a test of $H_{0}:\mu_{1}=\mu_{2}$ versus $H_{a}:\mu_{1}\neq \mu_{2},$ where $\mu_{1}$ and $\mu_{2}$ denote the respective means of the populations. Assume we have two functional datasets $s:=\{s_{1},\ldots ,s_{n}\}$ and $u:=\{u_{1},\ldots ,u_{m}\}$ respectively from populations $\Pi_{1}$ and $\Pi_{2};$ both of functions supported on a compact interval I. The test uses the statistic $T:=\int_{I}{\left(\bar{s}\left(t\right)-\bar{u}\left(t\right)\right)}^{2}dt,$ where $\bar{s}$ denotes the sample mean of $\left\{s_{1},\ldots ,s_{n}\right\}$ and equivalently for the $\bar{u}.$ Here we use a non-parametric test, using bootstrap to emulate the distribution of the data. Thus, it is computed *B* bootstrap samples from *s* and independently from u. For b=1,…,B, we denote these bootstrap samples by $\left\{s_{b,1}^{*},\ldots ,s_{b,n}^{*}\right\}$ and $\left\{u_{b,1}^{*},\ldots ,u_{b,m}^{*}\right\}$ and the respective bootstrap statistic under the null hypothesis by $T_{b}^{*}:=\int_{I}{\left({\bar{s}}_{b}^{*}\left(t\right)-\bar{s}\left(t\right)-{\bar{u}}_{b}^{*}\left(t\right)+\bar{u}\left(t\right)\right)}^{2}dt,$ where ${\bar{s}}_{b}^{*}$ is the sample mean of $\left\{s_{b,1}^{*},\ldots ,s_{b,n}^{*}\right\}$ and equivalently for ${\bar{u}}_{b}^{*}.$ Then, the *p*-value of the test is computed as $\left(1+\sum_{b=1}^{B}I\left(T^{*}b\geq T\right)\right)/\left(B+1\right)$ where I(·) is the indicator function that takes value one if $T^{*}b\geq T$ and zero otherwise. Here we use B=999.

### 3.2. Results of Applying the Tests to the Real Data

We apply the hypothesis tests to the sample $a_{i},$ i=1,…,187, assuming it is a simple random sampling. Forty-one of these elements are accelerations belonging to the group of patients in the early-stage of the disease, 100 to the middle-stage and 46 to the late-stage. We consider the different curves as entities and not the patients due to only having a set of 35 patients (seven in early-stage, 18 in the middle and 10 in the late) and therefore having a small sample size for the early-stage patients. Having a sample size not large enough may result in not having evidence to reject the null hypothesis when if it is not true. We do the study separately for the x, *y* and *z*-axis.

#### 3.2.1. Results of Applying the ANOVA Tests

**Figure 3:**
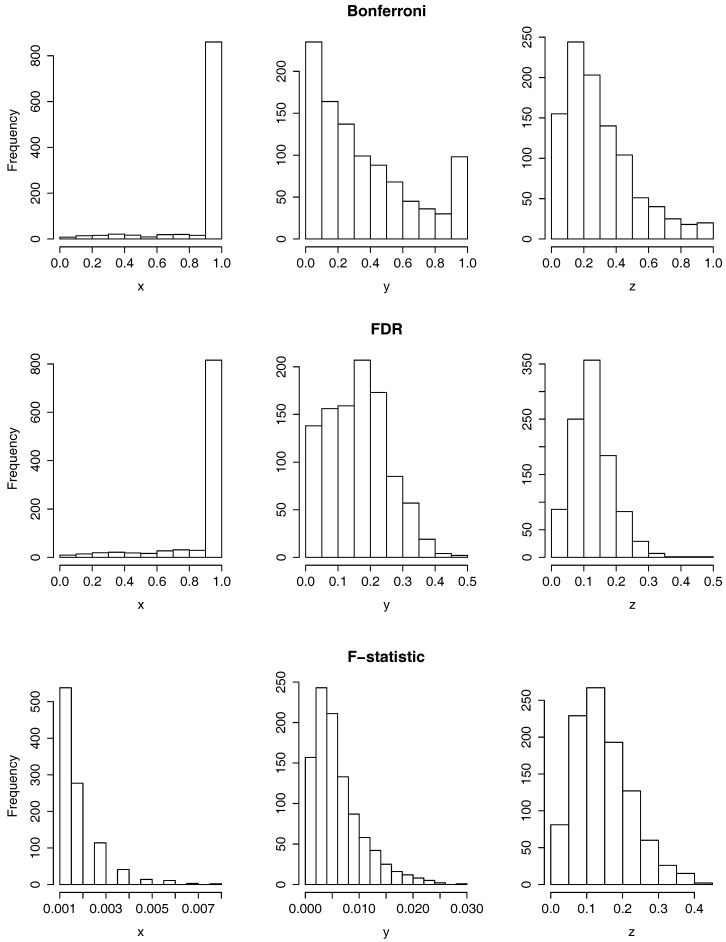
Histograms of 1000 *p*-values resulting from applying the two functional ANOVA tests based on random projections: based on the Bonferroni correction (**top row**) and on the FDR (**middle row**) and the functional ANOVA based on the F-statistic (**bottom row**).

In Figure 3, we can observe the histograms of the *p*-values resulting from testing the null hypothesis $H_{0}:\mu_{1}=\mu_{2}=\mu_{3}$ using the two functional ANOVA tests based on random projections (the one that uses the Bonferroni correction in the first row and the one that uses the FDR in the second) and the functional ANOVA based on the maximum of the F-statistic, in the third row. As we perform each of the three tests separately for the x, *y* and *z*-axis, the figure has three columns corresponding each to one of the three axis. If all the elements of the sample had been measured in the same time domain, we would have computed a total of six *p*-values, one per test and coordinate axis. As explained in the section above, however, we run each of the six cases for j=1,…,1000, applying for each *j* the test to a data sample where each curve $a_{i}$ is recorded at times $\{1+l_{i}\left(j\right),\ldots ,l_{i}\left(j\right)+l\},$ i=1,…,n.

It is clear from the last row of Figure 3 that there is a rejection of the null hypothesis when the functional ANOVA test based on the maximum of the F-statistic is applied on the *x*-axis and when is applied on the *y*-axis, as all the *p*-values are below 0.03, which is below the significance level of 0.05. On the contrary, it is observed from the first column of Figure 3 that there is little or no rejection of the null hypothesis when the two tests based on random projections are applied to the *x*-axis. To summarize the results of the *p*-values for a more detailed study, in the first three columns of Table 1 it is displayed the mean and standard deviation of the *p*-values per each hypothesis test, labeled as Bonferroni, FDR and F-statistic, and coordinate axis.

Furthermore, in Table 1, it is shown the proportion of *p*-values that are smaller or equal than 0.05, among the 1000. To analyze these results, it should be noticed that, under some independency assumptions, when a null hypothesis is true and it is tested a multiple number of times, it is expected to be rejected 5% of those times, at significance level 0.05. The reason for this is that the *p*-values follow a uniform distribution on [0,1] when the null hypothesis is true. Taking this into account, it is clear that there exists no evidence to reject the null hypothesis when the two tests based on random projections are applied to the *x*-axis (first two columns of Table 1: Bonferroni and FDR) as the proportion of *p*-values smaller or equal than 0.05 is 0.004. On the contrary, when the test based on the F-statistic is applied on the *x* and *y* coordinate axis, all the *p*-values are larger than 0.05. In the other five cases, when restricted to the first three columns of the table, the proportion of *p*-values smaller than, or equal to 0.05 is moderate, between the 6.5% and the 13.8% percentage. Although this is higher than the 5% of the cases, it would be better to have a test-statistic that provides a much higher proportion of rejections of the null hypothesis, so that we are certain that the null hypothesis has to be rejected. For a more detailed explanation, see below in the conclusions of the results.

#### 3.2.2. Results of Applying the Equality of Means Tests

**Figure 4:**
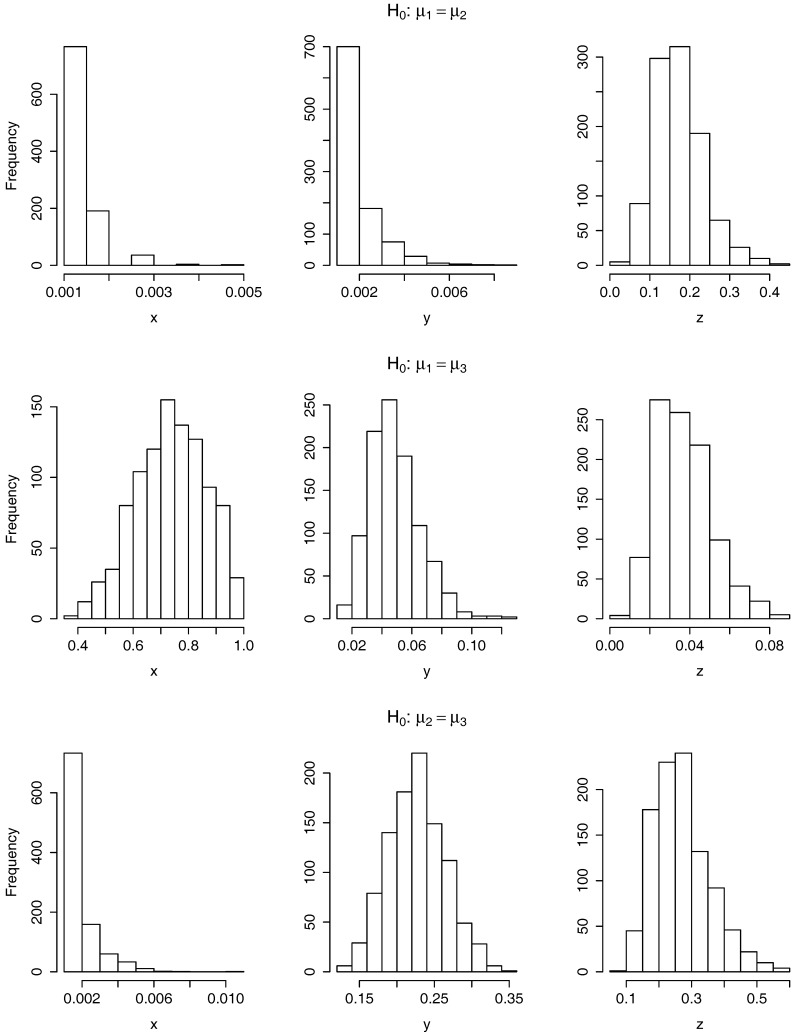
Histograms of 1000 *p*-values resulting from applying the test of equality of means based on the $L_{2}$ norm for the null hypothesis: $H_{0}:\mu_{1}=\mu_{2}$ (**top row**), $H_{0}:\mu_{1}=\mu_{3}$ (**middle row**) and $H_{0}:\mu_{2}=\mu_{3}$ (**bottom row**) on the *x*-axis (**left column**), *y*-axis (**middle column**) and *z*-axis (**right column**).

In Figure 4, we have displayed the six histograms of 1000 *p*-values resulting from testing the null hypothesis $H_{0}:\mu_{1}=\mu_{2}$ (top row), $H_{0}:\mu_{1}=\mu_{3}$ (middle row) and $H_{0}:\mu_{2}=\mu_{3}$ (bottom row) on the *x*-axis (left column), *y*-axis (middle column) and *z*-axis (right column). As when using the ANOVA tests, we compute here 1000 *p*-values per test due to for all the curves to have the same time domain we restrict at random the time domain of each curve to the one with smallest time domain. It is clear from the histograms that the null hypothesis is rejected the 1000 times when testing $H_{0}:\mu_{1}=\mu_{2}$ on the *x* and *y*-axis and when testing $H_{0}:\mu_{2}=\mu_{3}$ on the *x*-axis. It is also clear that the null hypothesis is rejected no time when testing $H_{0}:\mu_{1}=\mu_{3}$ on the *x*-axis and when testing $H_{0}:\mu_{2}=\mu_{3}$ on the *y* and *z*-axis.

For three other cases, it is helpful to observe the last three columns of Table 1 where, as for the previous tests, we have included a summary of the *p*-values for each of these six tests. There is displayed the mean and standard deviation of the *p*-values and the proportion of *p*-values smaller or equal than 0.05. For $H_{0}:\mu_{1}=\mu_{2}$ on the *z*-axis we obtain that the proportion of rejections is 0.005. As explained above, as this value is smaller than 0.05, we consider that there is no enough evidence to reject $H_{0}:\mu_{1}=\mu_{2}$ on the *z*-axis. On the contrary, when testing $H_{0}:\mu_{1}=\mu_{3}$ we are able to reject 58.8% of the times on the *y*-axis and 83.3% on the *z*-axis.

#### 3.2.3. Conclusions on the Results of Applying the Tests

We consider that the no rejection of the null hypothesis when applying the tests based on random projections happens because these tests assume the Gaussianity of the data. Furthermore, the no absolute rejection in any of the tests applied on the *z*-axis is due to the difference in accelerations among the patients occur in the xy-plane, which is the plane in which they move. Note that no rejecting the null hypothesis does not imply accepting it but that there is no evidence to reject it. Rejecting the null hypothesis, however, does imply accepting the alternative.

The above analysis on the test of equality of means is relevant in the sense that explains that it is relatively easy to distinguish:

- The patients in the early stage of their disease from the middle stage because of the *x* and *y*-axis.
- The patients in the middle stage of their disease from the late stage because of the *x*-axis.

Furthermore, it is harder, but possible, to distinguish:

- The patients in the early stage of their disease from the late stage because of the *y* and *z*-axis.

This analysis is consistent with our findings in Section 4 where using the neural network classifier we misclassified a patient of the late-stage.

### 3.3. Functional Supervised Classification

The aim of this section is to apply a functional classifier to the Alzheimer data. A functional classifier differs from a multivariate classifier in that it considers the given data as functional. The classifier we apply here is proposed in [8] and is based on the functional statistical depth [9] and a multivariate non-parametric kernel classifier. This multivariate classifier uses the euclidean norm and performs a non-parametric estimation of the density function of each of the three stages of the disease through the use of the well-known Nadaraya-Watson estimator. The functional statistical depth of the data is computed with respect to each of the three groups (early, middle and late-stage), living in a three-dimensional space. Thus, the functional data are reduced to $R^{3}$ through the depth and the multivariate classifier is applied to these three-dimensional depth values.

The functional statistical depth of an element of a space with respect to a distribution of probability is, informally speaking, a measure that shows how deep is the element of the space with respect to the distribution. The functional depth we use here is the *h*-mode depth [10] and has the characteristic of giving a higher depth value to the mode of the distribution, assuming it is unique. Other depth functions give a higher value to the median of the distribution. We use here the *h*-mode depth because of the nice properties it satisfices according to [9].

As commented at the beginning of the section, a preprocess of the data is required before any functional analysis is performed for all the curves to have the same length. This preprocess, which has a random component, is perform 10 times; and in each of those 10 times the same below procedure is undertaken. We run it here 10 times instead of the 1000 times of Section 3.1 and Section 3.2 due to the high computational cost of the functional supervised classification.

The data consist of a sample of 35 patients. Although it is common in a supervised classification analysis to split it into a training and test sample with, for instance, a percentage of the data of approximately 20–80%, here we consider as test sample only one patient and the remaining 34 patients as training sample. We do it for each of the 35 elements of the sample. The reason for doing so here is that this scenario should provide the best success rates. We like to be here under the best case scenario because, as we see later in the paper, the functional classifier on the Alzheimer data can be improved by other type of classifier.

When classifying a particular patient with respect to the other 34 patients, we classify all the curves that entitle this test patient. Thus, the patient gets associated the group that the majority of curves got associated. As the procedure is run for 10 times, we associate to the patient then, the majority group over the 10 times. The results are illustrated in Table 2. We do it separately using only the information on the *x*-axis and on the *y*-axis and then with the three axis altogether. The reason for that is that the movement of the patients is on the xy-axis.

From the 35 patients, 7 belong to the early stage, 18 to the middle and 10 to the late stage. Taking this into account, it can be observed from Table 2 the high misclassifications for each of the three stages of the disease and its associated low success rate. Particularly, it is clear the impossibility of this classifier of classifying correctly the patients in the early and late stage of the disease. It is straight forward from the displayed of the success rates that it would have been almost equivalent to associate the patients at random to a class. Therefore, a better classifier is required. We explore this option in the next section. 

## 4. Supervised Learning Classifiers

Supervised learning is a machine learning methodology whose goal is to infer a function from a labeled data set commonly called the training set. The training data consist of a set of training examples. Each example is a pair consisting of an input object (a vector of features) and an output value (the label). A supervised learning algorithm takes as input the training data and produces as output the desired function which can be used for predicting the labels on new examples. In this section, we train a neural network that identifies movement patterns of Alzheimer’s patients relating these patterns with the stage of the disease. For this purpose, we observe free movement of the patients not conditioned to the performability of specific exercises or tasks using the accelerometer of the smartphone and no other specific device. We also compare the performance of our neural network with other supervised learning classifiers.

### 4.1. Filtering Features

We consider the lack of homogeneity of the time frames (see Section 2). For this reason, we add separately the minutes and s to generate the data. This procedures is carried out as follows. If we have $o_{i},$ and $s_{i}$ is the limit of $i_{t_{i}}$ to s and $m_{i}$ to min. For instance, given $i_{t_{i}}=800.21$ s, then $s_{i}=801$ s and $m_{i}=14$ min. This procedure is carried out with each of the three axis of $o_{i}$ and each $t\in \{1,\ldots ,s_{i}\}$. The sum, median and mean of the accelerations are calculated at timestamps $\{T\in \left\{i_{1},\ldots ,i_{t_{i}}\right\}:t-1<T\leq t\};$ and for each $t\in \{1,\ldots ,m_{i}\}$ the sum, median and mean of the accelerations are also calculated at timestamps $\{T\in \left\{i_{1},\ldots ,i_{t_{i}}\right\}:t-1<T/60\leq t\}.$

In general, the $o_{i}$ do not have the same time length and, in other words, they are in different dimension. This involves an additional difficulty to use supervised classification techniques. The classification techniques filter the features of the data that enable us to find differences among the entities whose data should be classified (the stage of the disease in our case study). Therefore, our first goal should be to identify discriminative features in the data and to perform the classification using these features. As consequence of filtering the same features from each dataset, the classification techniques process always sets of the same dimension. For this, each $o_{i}$ is processed to calculate the sum, mean, median, minimum and maximum of the accelerations. A total of fifteen real random variables are generated when s are added and other fifteen variables with minutes for each of the dimensions of $R^{3}$.

The curves that represent the acceleration also provides useful information by means of integrating them along time, first, calculating the speed, and second, calculating the space. Thus, using the trapezoidal integration method, we generate 90 real random variables for each of the dimension of $R^{3}.$ Therefore, we identify the data generated with this integration by $O_{1},\ldots O_{n},$ where $O_{i}:=\left(X_{i},Y_{i},Z_{i}\right),$ with $X_{i}\in R^{90},$ $Y_{i}\in R^{90},Z_{i}\in R^{90},$ for all i=1,…,n.

### 4.2. Neural Networks Versus other Classifiers

Next, we use the dataset to build an artificial neural network. Additionally, we apply other machine learning classifiers and show how the success rate obtained with the neural network has no parallel. To this end, we divide the dataset into training and test. For the splitting, we propose to divide the sample of 187 elements into approximately 80% for training and 20% for testing. It is approximately because we have a total of 187 data and 35 patients. Thus, if an element of the sample falls into the training sample, the rest of elements of the sample that belong to the same patient automatically falls into the training sample. The split of the sample into training and test is performed at random.

The split we study in this section consists of a test sample of size thirty-four, and six patients (1 early-stage, 3 middle-stage and 2 late-stage), and a training sample of size 153, and 29 patients (6 early-stage, 15 middle-stage and 8 late-stage). This is shown in Table 3 after the row named studied. The notation used in what follows is $O_{t_{1}},\ldots ,O_{t_{r}}$ with r=153 for the training sample and $O_{T_{1}},\ldots ,O_{T_{e}}$ with e=34 for the test sample . In other to show how other splittings are possible. In Table 3 it is displayed the obtained results of performing the splitting two more times. In the first of this cases, the test sample, of size thirty-eight, is constituted by 7 patients (2 early-stage, 3 middle-stage and 2 late-stage) and the second is of size 39 and constituted by 8 patients (2 early-stage, 4 middle-stage and 2 late-stage).

**Figure 5:**
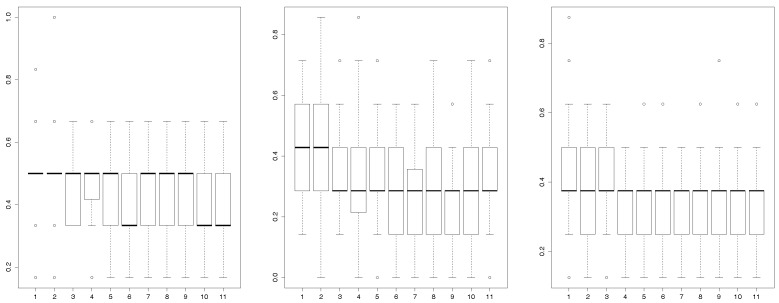
Boxplot of the ercentage of misclassified patients. The test data corresponding to the splitting, on training and test sample, under study (**left**), to Splitting 1 (**middle**) and to Splitting 2 (**right**).

A randomly procedure uses each training sample to generate one hundred neural networks for eleven different layers [11]. These different layers are represented on the *x*-axis of the Figure 5. Left panel of Figure 5 shows the rate of misclassifications that the neural networks generate when they are applied to the test sample. Once each of the $O_{T_{1}},\ldots ,O_{T_{e}}$ are classified in its corresponding stage, we classify the patients associated to them using the majority vote rule.

According to the sample of this study, the most suitable network misclassifies 2 patients (included in the test sample) for the third layer. However, only 1 patient is misclassified by this neural network for the other layers, an 83% success rate. The results of these tests (83% success rate ) show that the neural network is a suitable tool to recognize the stage of the disease in a context of variability in which the participants have the ability to perform different actions and no specific protocol is used to avoid accidentally changes on the orientation of the smartphone, including possible different placements of the smartphone. Moreover, the box-plot (see left panel of Figure 5) shows that the most suitable layers to reduce the misclassifications are the layers number six, ten and eleven. Table 4 specifies the parameters of the neural network chosen. Note that here we do not need to directly focus on the properties of the estimators such as using an uniformly minimum variance unbiased estimator, due to the classification methodology performed is based on artificial neural networks, otherwise known as a *black-box*.

This study was complemented using two additional splittings. The most suitable neural network misclassifies a patient with the layers number one and three in the Splitting 1. However, the other layers do not generate any misclassifications (see the middle panel of Figure 5). Moreover, the most suitable neural network generated by the Splitting 2 misclassifies patients in all the layers (see right panel of Figure 5). The box-plots (see middle and left panel of Figure 5) evidence that the most suitable layers to be selected are the number nine for Sample 1 and the number four or seven for Sample 2.

The R language has been used to implement the computational support that builds the neural network (package *neuralnet*) and to experiment with three new machine-learning classifiers. Thus, we can compare the results of the neural network with other machine-learning procedures. These new classifiers are Support Vector Machines (package *rpart* of R), Random Forest (package *randomForest* of R) and Random Trees (package *rpart* of R). Table 5 shows that an early-stage patient and two late-stage patients are always misclassified in these new experiments. We can conclude that these new techniques classify properly the middle-stage class that includes the highest number of patients and a larger amount of accelerometry data. However, these new experiments evidence the problems to classify patients of the other classes.

## 5. Related Work

In the healthcare field, the relationship between cognitive decline and movement disorders, specially gait disorders, is well known. Distinctive impairments in gait parameters have been described in patients with dementia, such as decreased walking speed, cadence, step-length and, more specifically, increased stride-to-stride and stride time variability [4]. Current evidence suggests a close relationship between gait and cognitive function, in particular executive function. Indeed, a gait study with trunk-accelerometers in patients with dementia and healthy elderly subjects found moderate to high correlations (r > 0.51) between executive tasks and gait parameters [4]. Brain imaging studies also support this theory [12], as the work of Nakamura [13], who found a significant association between decreased frontal lobe blood flow in patients with Alzheimer’s disease (AD) and increased stride length variability.

Growing evidence also shows that patients with dementia exhibit gait abnormalities even since early stages. A recent meta-analysis showed that poor gait performance predicted a subsequent development of dementia even years before the onset of cognitive symptoms [14]. This work gathered 12 articles including mostly healthy subjects, in addition to subgroups of patients with mild cognitive impairment in two works and a subgroup of patients with Parkinson’s disease without cognitive impairment in one work. These subjects underwent a gait assessment by clinical examination, gait speed and, only in two works, an analysis of spatiotemporal gait parameters by a pressure-sensitive electronic surface. After a follow-up of 3–9 years to determine the incidence of dementia, a pooled hazard ratio of 1.53 (*p* < 0.001) was found for any kind of dementia. Likewise, gait disturbances were also found to be a stronger predictor of non-AD dementia (as vascular dementia) than for AD dementia.

Furthermore, it has been proposed that a quantitative analysis of movement parameters may offer more information than clinical examination alone. Another study published in 2016 [15] found that abnormalities of gait worsened over time in parallel to the progression of cognitive decline. 1719 subjects, including healthy elderly volunteers and patients with mild cognitive impairment, AD dementia and non-AD dementia were assessed by a GAITRite System, a pressure-sensitive electronic surface which provides spatiotemporal gait parameters, such as stride length, stride time, swing time, single support time, etc. The results showed a progressive impairment of the spatiotemporal gait parameters from healthy elderly to moderate dementia, as well as a progressive increase in the magnitude of the effect sizes of the various parameters. Again, a greater decline was observed in patients with non-AD dementia than in patients with AD.

Thus, it is clear that patients with dementia show gait abnormalities since early stages and these disturbances probably depend on the stage and the type of underlying disease. Therefore, a proper evaluation can offer clinically relevant information in terms of diagnosis, early detection and assessment of the stage of the disease. Several research works have been conducted regarding this issue and the use of portable triaxial accelerometers. As an additional advantage, accelerometers allow the conduction of field studies, as they are small devices that do not require continuous supervision. This means that the study can be prolonged many hours or even days, which offer a greater amount of available data. Likewise, they are carried out in non-supervised settings and by means of unobtrusive devices, which helps the patient to pay less attention to the process and so to obtain a more realistic behavior, given that the possibility of the explorer’s influence on the performance is greater in supervised assessments.

On the other hand, the disadvantage of field studies is that the performance usually lacks an external reference. Therefore, when an specific gait analysis is desired, an additional process to identify gait episodes from the accelerometer data is required. An example of such methodology is described by Gietzelt et al. [16] in a study that analyzed the data captured by a single waist-mounted accelerometer in 10 patients with dementia and 10 active older people during seven days. Using the identified gait parameters, it was possible to distinguish between demented patients and healthy older people with an accuracy of 89.2% and most gait parameters (specially compensation movements and variance of the accelerometric signal) showed high area under the curve values. Based on this system, the same authors conducted a longitudinal cohort study [17] to make a fall prognosis in patients with dementia. Forty patients from a nursing home were included and each participant wore a triaxial accelerometer for one week during four different visits. The falls occurred during the study were combined with gait parameters for the construction of a decision tree to determine if the patient was at risk of suffering a fall. It showed a rate of correctly classified gait episodes associated to fallers of 74.8% for a period of 4 months with acceptable sensitivity (78.2%) and specificity (71.2%), so the authors conclude that it is possible to make a fall prognosis in patients with dementia by the use of accelerometry.

Accelerometry has also been employed with the aim of comparing movement disturbances between cognitive disorders and other neurological diseases, as Parkinson’s disease (PD). Yoneyama et al. [18] studied 13 healthy subjects, 26 PD patients, 13 patients with MCI and 13 patients with dementia, who wore a single trunk-mounted accelerometer for 24 h in an unsupervised setting. An analytical algorithm was applied to the provided data to develop a set of new parameters which showed that each group of patients displayed a characteristic movement pattern that could differentiate it from the other groups.

However, accelerometry has not only been used to analyse instrinsic movement features, but also to study other dementia-related symptoms, as changes in motion behaviour. Kirste et al. [19] studied everyday motion behaviour in 23 AD patients without major clinical behaviour abnormalities and 23 healthy partners by ankle-mounted three-axes accelerometers, with an average recording duration of 53.4 h. They found differences in behavioural motion features that could correctly discriminate between patients and healthy subjects with an accuracy of 91% and were significantly correlated with CMAI (Cohen-Mansfield Agitation Inventory) scores, a widely used scale to assess behavioural changes. Not too many studies based on the accelerometers integrated in smartphones have been carried out, as the work of Capela [20], which studied human activity recognition in both able-bodied subjects and patients who had suffered a stroke. To fill this gap, this work has experimented with a methodology that takes as input data provided by accelerometers of smartphones of Alzheimer’s patients and outputs an analysis of the relationship between movement disturbances and the stage of the disease.

## 6. Conclusions

This paper uses functional data analysis and machine learning techniques to perform an analysis on three-dimensional functional data from Alzheimer’s patients. Functional data are regarded as observations of continuous data measured on a grid. The studied functional data are the accelerations recorded by an accelerometer device of android smartphones. These data are complex in certain ways:

1. There is a range of observed three-dimensional functional data per patient, which varies between two and eight.
2. Each functional datum is observed in a different grid, with a different frequency of observations, even when the data belong to the same patient.
3. Each functional datum is observed in a domain of different length.

To the best of our knowledge, this paper is the first one to handle data with this type of complexity, particularly because of the domains of different length. We deal with it performing a preprocess of the data, which differs when applying a functional data analysis and when machine learning techniques.

The preprocess for the functional data analysis makes use of the fact that the set of data regarding each patient is in essence a set of observations of a stationary random process. Thus, the observations are equally distributed if they are translated in time. Considering this, we select at random a section of each functional datum whose domain length is equal to the smallest. Then, we apply the required technique:

- Hypothesis testing on the equality of the means of groups of patients on different stages of the disease to find differences on the accelerations patterns among different stages of the disease.
- Functional supervised classification to classify the patients according to their set of observed acceleration curves into their stage of the disease.

The novel real data used in this paper consist of 187 three-dimensional functional data belonging to a total of 35 patients. Making use of different functional hypothesis tests, we generally reject the null hypothesis of equality of means of the accelerations for the different stages of the disease. When functional classification procedures are applied to the data, however, we do not obtain a triumphant success rate. We use then machine learning techniques.

Machine learning techniques require of a set of multivariate data. Thus, we preprocess the data so that we summarize it into a series of random variables such as the mean, median, minimum, maximum, etc., among a total of ninety random variables. Among the applied machine learning techniques, the best obtained results are for the artificial neural network. We obtain a success rate of the 83% percent, which is outstanding for this type of complex data.

We have conducted this study as a first approach to investigate whether the proposed methodology possesses some potential ability to correctly classify patients with cognitive impairment by means of their mobility patterns. The findings of our work lead us to conclude that it seems possible. Here, this methodology has been employed to characterize the stage of the disease in patients with Alzheimer’s disease, but it may be adapted to other multiple clinical applications, such as monitoring nightly wandering or abnormal motor behaviour. It could also be applied with diagnostic aims to contribute to differential diagnosis processes between different types of dementia, or in mild cognitive impairment, a situation where it is uncertain whether it is going to evolve to dementia or it is going to remain stable. This approach constitutes an inexpensive and easy-to-use resource which could enable caregivers to provide medical useful information. Further research work is needed to replicate these results in wider samples of patients and to test its utility for other clinical purposes. These future works should be a set of comparative experiments that contrast the advantages and disadvantage of our current approach against experiments with less degree of variability in which the patients perform very controlled activities in a laboratory and the accelerometer is a device located on the body of the patient, including the use of a variety of different types of smartphone with accelerometer. In particular, our group would like to extend the proposed methodology to a sample of patients with mild cognitive impairment to test whether it is possible to identify subjects with mild cognitive impairment due to Alzheimer’s disease, which equals a pre-dementia stage and represents a major medical challenge.

## Figures and Tables

**Table 1:** Mean, standard deviation and proportion of values smaller or equal than 0.05 of 1000 *p*-values resulting of applying separately for each coordinate axis, x, *y* and z, the hypothesis tests: functional ANOVA tests based on random projections using the Bonferroni correction (first column) and the FDR (second column), functional ANOVA test based on the F-statistic (third column) and functional test of means based on the $L_{2}$ norm for contrasting the acceleration curves corresponding to patients in the early-stage of the disease against the middle (fourth column), the early against the late (fifth column) and the middle against the late (sixth column).

	Tests					
	Bonferroni	FDR	F-Statistic	$H_{0}:\mu_{1}=\mu_{2}$	$H_{0}:\mu_{1}=\mu_{3}$	$H_{0}:\mu_{2}=\mu_{3}$
*x*-axis						
mean	0.9266	0.8957	0.0018	0.0013	0.7356	0.0020
stand. dev.	0.2027	0.2060	0.0011	0.0006	0.1256	0.0012
proportion	0.004	0.004	1	1	0	1
*y*-axis						
mean	0.3603	0.1616	0.0066	0.0021	0.0492	0.2267
stand. dev.	0.3015	0.0931	0.0046	0.0012	0.0168	0.0381
proportion	0.128	0.138	1	1	0.588	0
*z*-axis						
mean	0.2971	0.1282	0.1465	0.1733	0.0377	0.2702
stand. dev.	0.2145	0.0620	0.0762	0.0616	0.0139	0.0853
proportion	0.065	0.087	0.081	0.005	0.833	0

**Table 2:** Misclassification and success rate obtained in the functional supervised classification when applied to the data in the *x*-axis, *y*-axis and xyz-axis.

	Missclassifications				Success Rate			
	Early	Middle	Late	Total	Early	Middle	Late	Total
*x*-axis	7	2	9	18	0%	89%	10%	49%
*y*-axis	7	4	8	19	0%	78%	20%	46%
xyz-axis	6	2	9	17	14%	89%	10%	51%

**Table 3:** Stages of the patients selected in the training and test sample for each of the performed splittings.

			Training Sample					Test Sample			
Splitting			Early	Middle	Late	Total		Early	Middle	Late	Total
studied			6	15	8	29		1	3	2	6
1			5	15	8	28		2	3	2	7
2			5	14	8	27		2	4	2	8

**Table 4:** Configuration of the neural network selected.

Neural Network Parameters	Values
Package	neuralnet
Input neurons	90
Hidden neurons	175
Output neurons	1
Bias	1 per hidden layer
Max iterations	1000
Activation function	logistic
Algorithm	resilient back-propagation with weight backtracking (rprop+)

**Table 5:** Experiments with other methods for supervised classification. NN stands for neural networks, RT for random trees, RF for randon forest, and SVM for support vector machine.

	Missclassification Rate				Success Rate			
Technique	Early-Stage	Middle-Stage	Late-Stage	Total	Early-Stage	Middle-Stage	Late-Stage	Total
NN	0	0	1	1	100%	100%	50%	83%
RT	1	0	2	3	0%	100%	0%	50%
RF	1	0	2	3	0%	100%	0%	50%
SVM	1	0	2	3	0%	100%	0%	50%
